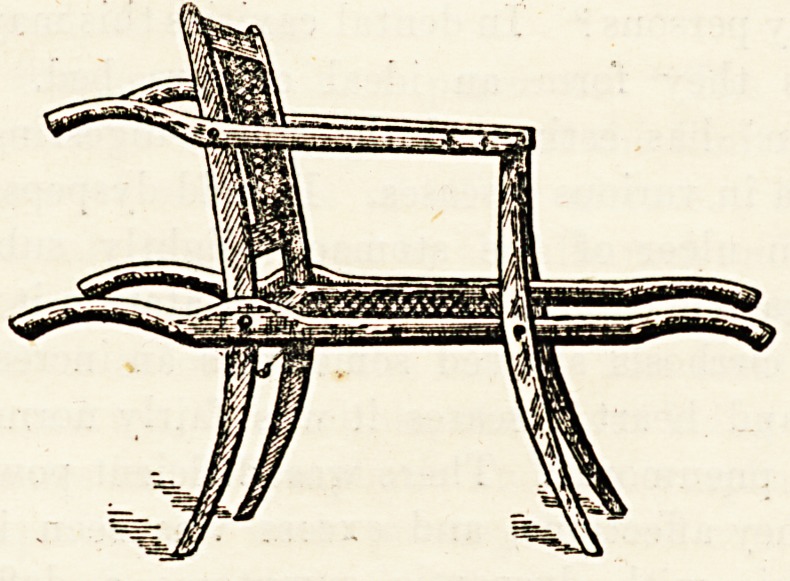# New Appliances and Things Medical

**Published:** 1900-12-01

**Authors:** 


### NEW APPLIANCES AND THINGS MEDICAL.

[We shall be glad to receive, at our Office, 28 & 29 Southampton Street, Strand, London, W.C., from the manufacturers, specimens of all new preparations and appliances which may be brought out from time to time.]

### FORMIC SULPHUGATORS.

(Sanitas Company, Limited, Bethnal Green, London, E.)

These new sulphugators are a modification of Kingzett's original patent, which it will be remembered was a special form of sulphur candle. The appliances under review are supplied in two forms which both embody the same principle: one the more expensive variety is rather more efficient, but at the same time rather more complicated in design. Roughly speaking, the Formic Sulphugator consists of a double tin: the inner chamber contains an ordinary sulphur candle provided with a lighter; in the outer chamber there is a preparation of para-formaldehyde with other inert material. Before use, water or a solution of formaldehyde should be poured on to the top of the contents of the outer chamber, and a light applied to the sulphur candle in the central compartment. The heat generated by the latter not only liberates sulphurous acid by the combustion of the sulphur, but at the same time causes an evaporation of formaldehyde. These two powerful antiseptic vapours in association with steam are set free into the air, and are capable of disinfecting empty rooms, stables, kennels, poultry houses, and the like in a manner which embodies the latest scientific principles of microbic destruction. Full directions are supplied with the apparatus, which will be found invaluable for domestic or institutional use.

### LIQUID TRAUMATOL, BIPALATINOIDS OF CREASOTE AND EASTON'S SYRUP AND BIPALATINOIDS OF HYPOPHOSPHITE AND CREASOTE.

(Oppenheimer, Son, and Co., Limited, 179 Queen Victoria Street, London, E.C.)

Traumatol is a powerful antiseptic, as one would expect from its chemical constitution, namely, iodide of cresylic acid. When diluted it may be employed with the greatest advantage as a gargle or an antiseptic lotion in surgical practice. The principle of the bipalatinoid is happily applied for the combination of the drugs above mentioned. As is well known, creasote interacts chemically with both phosphates and hypophosphites, and therefore they cannot exist together in the same preparation unless separated by some mechanical means as that provided by the double compartments of the bipalatinoid. The administration of these two drugs in a form in which they can each separately influence the tissues according to their specific action is a distinct advance in artistic medication.

### FOLDING CARRYING CHAIR.

(Farmer, Lane, and Co., 77 and 79 New Oxford Street, London, W.)

Many an invalid who would be thankful for a carrying chair is debarred from the comfort and convenience which this contrivance affords by the apparently trivial—but in small houses very practical—difficulty that no place can be found in which to put it. As usually made, it certainly is an awkward bit of furniture, and one which is apt to be in the way of everybody's shins. Messrs. Farmer, Lane, and Co., however, have got over this difficulty in the simplest way in the world, by just making the whole affair shut up, something after the manner of a deck chair. Nothing could be neater or more effectual. The chair they have built is perfectly firm and strong, there are no folding joints in the part by which the weight is carried, yet when not wanted the back and legs and arms all fold together, so that, like a deck chair, it can be reared up against the wall out of the way of everyone. Certainly a very useful application of a well-known principle.

## Figures and Tables

**Figure f1:**